# Associations between estimated fatty acid desaturase activities in serum lipids and adipose tissue in humans: links to obesity and insulin resistance

**DOI:** 10.1186/1476-511X-8-37

**Published:** 2009-08-27

**Authors:** Eva Warensjö, Magdalena Rosell, Mai-Lis Hellenius, Bengt Vessby, Ulf De Faire, Ulf Risérus

**Affiliations:** 1 Department of Public Health and Caring Sciences, Clinical nutrition and metabolism, Uppsala University, Uppsala, Sweden; 2 Department of Biosciences and Nutrition, Karolinska Institutet, Stockholm, Sweden; 3 Department of Medicine, Clinical Epidemiology Unit, Karolinska Institutet, Stockholm, Sweden; 4 Institute of Environmental Medicine, Unit of Cardiovascular Epidemiology, Karolinska Institutet, Stockholm, Sweden

## Abstract

Fatty acid composition of serum lipids and adipose tissue triacylglycerols (AT-TAG) partly reflect dietary fatty acid intake. The fatty acid composition is, besides the diet, also influenced by desaturating enzymes that can be estimated using product-to-precursor fatty acid ratios. The interrelationships between desaturase indices derived from different serum lipid fractions and adipose tissue are unclear, as well as their associations with obesity and insulin resistance. We aimed to investigate cross-sectional correlations between desaturase indices as measured in serum lipid fractions (phospholipids; PL and free fatty acids; FFA) and in adipose tissue (AT-TAG). In a population-based sample of 301 healthy 60-year-old men various desaturase indices were assessed: stearoyl-CoA-desaturase (16:1n-7/16:0; SCD-16 and 18:1n-9/18:0; SCD-18, respectively), delta-6-desaturase (20:3n-6/18:2n-6; D6D) and delta-5-desaturase (20:4n-6/20:3n-6; D5D). Correlations with BMI and insulin resistance (HOMA-IR) were also examined. SCD-16 and D5D were significantly correlated between fractions and tissues (all r > 0.30), whereas SCD-18 and D6D were not. Desaturase indices in serum FFA and AT-TAG were significantly correlated; SCD-16 (r = 0.63), SCD-18 (r = 0.37), and D5D (r = 0.43). In phospholipids, SCD-16 was positively correlated to BMI (r = 0.15), while D5D negatively to both BMI (r = -0.30) and HOMA-IR (r = -0.31), all p < 0.01. D6D in both phospholipids and AT-TAG was positively correlated to HOMA-IR and BMI (all p < 0.01). In conclusion, SCD-1 and D5D activity indices showed overall strong correlations between lipid pools. SCD-1 activity index in adipose tissue is best reflected by 16:1/16:0-ratio in serum FFA, but associations with obesity and insulin resistance differ between these pools. D5D in PL was inversely related to obesity and insulin resistance, whereas D6D index showed positive associations.

## Findings

The fatty acid composition of serum lipids and adipose tissue triacylglycerols reflect the dietary fat intake over the previous weeks and years, respectively [1-4]. The fatty acids composition is also influenced by desaturating enzymes and lifestyle factors such as smoking and physical activity [1,5]. Free fatty acids (FFA) are mainly released from adipose tissue by lipolysis and provide energy to a number of organs and serve as substrate for re-esterification of triacylglycerols [6]. Serum fatty acids and desaturase indices have been related to obesity [7], insulin resistance [8], type-2-diabetes [9] and the metabolic syndrome [10]. Desaturase indices, estimated as product-to-precursor fatty acid ratios, are employed in epidemiological studies. However, little data exist on how these indices relate to each other between different lipid pools and tissues. Accumulating evidence suggest that Stearoyl-CoA-Desaturase (SCD-1) enzyme activity may be regulated differently in adipose tissue and liver, and its link with insulin resistance and obesity may differ between these pools. The primary aim of the present study was to investigate associations between desaturase indices in serum phospholipids, FFA and adipose tissue triacylglycerols (AT-TAG). Secondary, associations between desaturase indices and risk markers were studied.

The study participants, 301 healthy men, were selected from a cohort of 60 year old Swedish men and women. The recruitment procedures and investigations have been previously described [11,12]. For the analysis of adipose tissue fatty acids a needle biopsy of subcutaneous adipose tissue was taken from the upper left buttock [13]. Blood samples were collected from the antecubital vein. Serum and adipose tissue samples were stored up to one year (-70°C) before analysis. The fatty acid composition of serum phospholipids, FFA and AT-TAG was measured as previously described [14]. Briefly, the samples were extracted in chloroform, separated by thin-layer -chromatography (this step was not carried out for AT-TAG), trans-methylated and then separated by gas liquid chromatography with a system from Hewlett-Packard (Avondale, PA)) on a capillary column (Quadrex, New Heaven, CT, USA). Methyl ester standards (GLC- 68A, Nu Check Prep, Elysian, MN, USA) were used to identify the different fatty acids. Desaturase indices were estimated according to the following: SCD-16: 16:1(n-7)/16:0, SCD-18: 18:1(n-9)/18:0, D6D: 20:3(n-6)/18:2 (n-6) and D5D: 20:4(n-6)/20:3 (n-6). Insulin resistance was estimated by HOMA-IR (fasting insulin (mU/l) × fasting glucose (mmol/l)/22.5) [15]. The statistical analysis was carried out with the software package STATA (version 8.2; STATA Corporation, TX, USA). Correlations were investigated with Spearman's rank correlation test and the test was significant at p < 0.05. No imputing for missing data was done.

**Figure 1:**
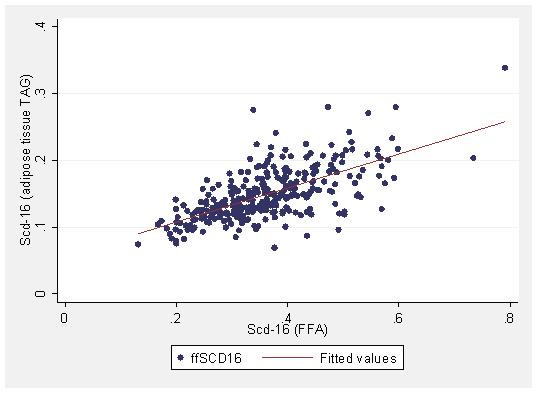
**Scatter plot showing the relation between SCD-16 (16:1/16:0) estimated in free fatty acids (FFA, x-axis) and adipose tissue triacylglycerols (TAG, y-axis)**.

The baseline characteristics of the study population are presented in Table 1. SCD-16 index was correlated between fractions and tissues and the strongest correlation was not surprisingly established between FFA and AT-TAG (Table 2 and the Figure 1). SCD-18 in free fatty acids was significantly correlated to both SCD-18 in phospholipids and AT-TAG. D5D was also correlated between tissues and fraction with the strongest correlation between FFA and AT-TAG. D6D in phospholipids and AT-TAG were correlated. SCD-16 index in phospholipids and AT-TAG was positively correlated to BMI, whilst SCD-16 in FFA was surprisingly negatively correlated to the HOMA-IR (Table 3). SCD-18 in AT-TAG was positively and in phospholipids negatively correlated to BMI and HOMA-IR. SCD-18 in FFA was also positively correlated to BMI. D5D in phospholipids were negatively associated with BMI and HOMA-IR, whereas D5D of AT-TAG was correlated to neither BMI or to HOMA-IR. D6D in both phospholipids and AT-TAG was positively correlated to HOMA-IR and BMI. The correlation between D5D in phospholipids and D6D in AT-TAG and HOMA-IR remained after adjustment for BMI.

**Table 1:** Baseline characteristics of the study subjects.

Baseline characteristics		N
Age (years)	63 (0.7)	301
BMI (kg/m^2^)	25.9 (3.1)	301
Total cholesterol (mmol/l)	5.9 (1.0)	301
LDL-cholesterol (mmol/l)	3.7 (1.0)	301
HDL-cholesterol^*a *^(mmol/l)	1.6 (1.4–1.8)	301
Triacylglycerol^*a *^(mmol/l)	1.1 (0.8–1.4)	301
Fasting insulin^*a *^(mU/l)	5.8 (4.2–8.0)	301
Fasting glucose^*a *^(mmol/l)	4.9 (4.6–5.2)	301
HOMA-IR^*a*^	1.3 (0.9–1.8)	300
Tobacco users (%)	23	301

Adipose tissue desaturase indices		

SCD-16	0.36 (0.1)	297
SCD-18	14.8 (3.6)	297
D6D^*a*^	17(14–20) × 10^-3^	286
D5D	2.5 (0.6)	286

Free fatty acid desaturase indices		

SCD-16	0.15 (0.04)	300
SCD-18	3.95 (0.73)	300
D6D^*a*^	33 (23–55) × 10^-3^	51
D5D^*a*^	3.1 (2.2–4.1)	51

Phospholipid desaturase indices		

SCD-16^*a*^	0.02 (0.017–0.023)	299
SCD-18	0.94 (0.12)	299
D6D^*a*^	0.15(0.13–0.18)	299
D5D	3.0 (0.71)	299

Mean and standard deviation are presented for normally distributed variables and median and interquartile range (25^th^–75^th ^percentiles) for skewed variables.

^*a *^Skewed variable

HOMA Homeostasis model insulin resistance

SCD Stearoyl-CoA-desaturase (SCD-16 = 16:1(n-7)/16:0, SCD-18 = 18:1(n-9)/18:0)

D5D delta-5-desaturase (20:4(n-6)/20:3 (n-6))

D6D delta-6-desaturase (20:3(n-6)/18:2 (n-6))

**Table 2:** Spearman rank correlations between estimated fatty acid desaturases in serum phospholipids, serum free fatty acids and adipose tissue triacylglycerols.

		Serum free fatty acids			Adipose tissue triacylglycerols		
		
		**SCD-16**			**SCD-16**		
		r	*P*	n	r	*P*	n
SCD-16	PL	0.54	0.000	299	0.39	0.000	295
	FFA	-	-	-	0.63	0.000	296

		**SCD-18**			**SCD-18**		
		r	*P*	n	r	*P*	n

SCD-18	PL	0.17	0.004	299	-0.05	0.44	295
	FFA	-	-	-	0.37	0.000	296

		**D6D**			**D6D**		
		r	*P*	n	r	*P*	n

D6D	PL	-0.13	0.44	39	0.53	0.000	295
	FFA	-	-	-	-0.06	0.69	48

		**D5D**			**D5D**		
		r	*P*	n	r	*P*	n

D5D	PL	0.31	0.03	50	0.36	0.000	284
	FFA	-	-	-	0.43	0.002	48

SCD Stearoyl-CoA-desaturase (SCD-16 = 16:1(n-7)/16:0, SCD-18 = 18:1(n-9)/18:0)

D5D delta-5-desaturase (20:4(n-6)/20:3 (n-6))

D6D delta-6-desaturase 20:3(n-6)/18:2 (n-6)

PL Serum phospholipids

FFA serum free fatty acids

TAG Triacylglycerols

**Table 3:** Spearman rank correlations between estimated desaturase activities in serum phospholipids and adipose tissue triacylglycerols and clinical variables.

		BMI			HOMA- IR		
		
		r	*P*	n	r	*P*	n
SCD-16	PL	0.15	0.009	299	0.08	0.17	298
	FFA	0.08	0.18	300	-0.16	0.006	299
	AT-TAG	0.17	0.004	297	-0.02	0.78	296

SCD-18	PL	-0.31	0.000	299	-0.41	0.000	298
	FFA	0.15	0.009	300	0.05	0.40	299
	AT-TAG	0.35	0.000	297	0.24	0.000	296

D6D	PL	0.36	0.000	299	0.32	0.01	298
	FFA	-0.13	0.37	51	-.0002	1.0	51
	AT-TAG	0.36	0.000	297	0.34	0.000	296

D5D	PL	-0.30	0.000	299	-0.31	0.000	298
	FFA	0.03	0.8	51	-0.16	0.27	51
	AT-TAG	0.03	0.6	286	-0.04	0.46	285

SCD Stearoyl-CoA-desaturase (SCD-16 = 16:1(n-7)/16:0, SCD-18 = 18:1(n-9)/18:0)

D5D delta-5-desaturase (20:4(n-6)/20:3 (n-6))

D6D delta-6-desaturase (20:3(n-6)/18:2 (n-6)

PL Serum phospholipids

AT TAG adipose tissue triacylglycerols

BMI body mass index

HOMA IR Homeostasis model of insulin resistance

### Correlations between desaturase ratios in FFA, PL and AT-TAG

This study confirms and extends information regarding desaturase indices in different lipid pools as well as in relation to obesity and insulin action. Estimated SCD-16 and D5D activities were both highly correlated between fraction and tissues, whereas D6D and SCD-18 was not. Associations between serum free fatty acids and AT-TAG for estimated SCD-16, SCD-18 and D5D, were strong and reflects the release of free fatty acids from adipose tissue into the circulation during lipolysis [16]. The correlation between serum free fatty acids and AT-TAG for estimated SCD-16 was the strongest, r = 0.63, p = 0.000. It is possible that the SCD-16 index in FFA to a large part reflect the SCD enzyme activity in adipose tissue, while estimated SCD in other serum fractions, cholesteryl esters and triacylglycerols, may mainly reflect liver SCD enzyme activity. This is useful information for future epidemiological studies that aim to examine adipose tissue SCD enzyme activity, and differentiate this index from the serum pools mainly reflecting liver SCD activities, i.e. cholesteryl esters, and triacylglycerols [17].

The use of SCD indices derived from phospholipids is however more unclear since there is significant of exchange of fatty acids within systemic circulation between lipid pools [17]. Further it is uncertain which tissue SCD index from phospholipids would reflect. Thus, interpretation of desaturation indices using phospholipids requires caution. To estimate SCD activity of the liver, triacylglycerol is in theory the optimal lipid fraction. Data on this fraction would have been useful to help understand the role of different lipid fractions and its link to liver versus adipose tissue, but were unfortunately not available in the present study. Analyses by Summers et al. [18] of 16:0 and 18:2 n-6 in platelet phospholipids, plasma cholesteryl esters, plasma triacylglycerols and plasma FFA however showed a strong concordance between plasma FFA and plasma TG. Such data suggest that several lipid fractions including FFA may reflect liver SCD1 to some extent. Although FFA at first glance should mainly reflect adipose tissue, it is possible that FFA may resemble a combination of adipose tissue SCD1 and liver SCD1, due to exchange of fatty acids between liver and adipose tissue.

It should further be noted that we have not controlled for the potential influence of dietary fatty acids on desaturation indices. Thus, although fatty acid ratios for estimating desaturation activities are meant to reflect enzyme activity other environmental factors including fatty acid intake may influence these ratios [5]. This study investigates the interrelationships between different desaturation indices, rather than exploring what different factors that may determine the level of these desaturation indices.

In the present study, the SCD-18 index in phospholipids and AT-TAG were not correlated. This might be due to endogenous processes such as the elongation of palmitic acid (16:0) to stearic acid (18:0) or the rapid conversion of 18:0 to oleic acid (18:1) [19], or that 18:1 is preferentially retained in adipose tissue [20]. Thus, a high SCD-18 index in serum phospholipids is more influenced by a diet high in 18:1 [21]. However, a high SCD-18 index in AT-TAG might be related to lipogenesis and therefore related to risk factors in the present study.

### Desaturase indices and risk factors

#### SCD-18 and SCD-16

The SCD index can be estimated as 16:1/16:0 or 18:1/18:0 and it is the former that has most consistently been related to metabolic complications [22]. When SCD-18 in phospholipids was used in the analyses it was negatively correlated to BMI and HOMA-index, while the relations between SCD-18 in AT-TAG and the same variables were positive, which are difficult results to interpret, but in analogy with the findings in a sub-group of the present study population by Sjögren *et al *[23]. In that study, the associations between desaturase mRNA expression and desaturase indices in adipose tissue and insulin resistance were investigated. Insulin-resistant subjects had a higher adipose tissue SCD-18, but not SCD-16, compared to insulin-sensitive subjects. This was also found in the present study. It was further suggested that desaturation indices in adipose tissue reflected the expression of SCD, but not of D5D or D6D in adipose tissue [23]. Neither D5D nor D6D indices in FFA correlated to risk factors in the present study, which is compatible with the results of Sjögren *et al *[23].

#### SCD-16, D6D and D5D

Previously, a high SCD-16 and D6D index was associated with obesity [7] and predicted the metabolic syndrome, associations that was confounded by obesity [10]. However, the D5D serum index was inversely related to obesity [7] and the development of the metabolic syndrome, but this association was independent of obesity [10]. A high D5D index in serum phospholipids and muscle has been related to enhanced insulin action in both Pima Indians [24] and Caucasian men [25]. Pan *et al*. [24] reported D5D index to be independently associated with obesity. The present results extend these previous observations and confirm that the SCD-16 index is primarily related to obesity, but not to insulin resistance, while the D5D index is independently related both to glucometabolic risk and obesity. D6D in phospholipids and AT-TAG were correlated to BMI and insulin resistance, which align with the notion that a high D6D index is associated with risk for the metabolic syndrome [10]. The negative association between SCD-16 in FFA and HOMA-IR is difficult to interpret and may be due to chance and requires further study.

### Possible mechanisms

Desaturases catalyze the synthesis of unsaturated fatty acids that are incorporated into cell membranes which thereby affect permeability and functional properties of cells. This may affect insulin signalling and receptor binding affinities. Besides, long chain polyunsaturated fatty acids synthesized by D5D and D6D may serve as ligands for transcription factors, such as sterol regulatory element binding protein1 and peroxisome proliferators activated receptors which interact with genes involved in lipogenesis and fatty acid oxidation. There is also a potential link between D5D and D6D activities and insulin resistance via inflammatory mediators, eicosanoids [20]. The SCD1 enzyme desaturates 16:0 and 18:0 as a direct result from *de novo *lipogenesis (DNL) or derived from the diet [17]. In the present population consuming a Western diet with a total fat intake of about 35% [12], the contribution of fatty acids from DNL is thought to be of lesser importance [26]. It is known that SCD1 activity is up-regulated in the liver in parallel to DNL on a high carbohydrate diet (75E%) but not at a high fat diet (40E%) [27]. Relationships between desaturases and insulin resistance may be directed in either direction; either disturbances in desaturases lead to metabolic changes or metabolic changes themselves lead to adaptive changes in desaturating enzymes in order to cope with an aberrant situation.

In conclusion, the SCD-1 and D5D indices showed strong correlations between lipid pools and tissues. We hypothesize that the SCD-16 index in FFA to a large part reflect the SCD enzyme activity in adipose tissue, while the SCD index in triacylglycerols and cholesteryl esters mainly reflect liver SCD enzyme activity. This is useful information to have when studying desaturase indices in epidemiological studies. SCD-16 in adipose tissue was related to BMI but not insulin resistance. D5D in phospholipids was inversely related to both these factors and D6D index showed positive associations.

## Competing interests

The authors declare that they have no competing interests.

## Authors' contributions

UR and EW conceived the study and interpreted the data. EW carried out the statistical analyses and drafted the manuscript. UR was also responsible for critical revision and supervision. MR has contributed to data collection and to critical revision of the manuscript. BW was responsible for the fatty acid analyses and interpretation of data and critical revision of the manuscript. MLH and UDF were responsible for data collection, handling of the database, interpretation of data, and critical revision of the manuscript. All of the authors have read and approved the final version of the manuscript.
